# Solitary metastasis of myxoid liposarcoma from the thigh to intraperitoneum: a case report

**DOI:** 10.1186/s12957-019-1724-3

**Published:** 2019-10-28

**Authors:** Dong-Wook Kim, Ye Seob Jee

**Affiliations:** Department of Surgery, Dankook University College of Medicine, 201 Manghyangro, Dongnam-gu, Cheonan, 31116 Republic of Korea

**Keywords:** Myxoid liposarcoma, Single metastasis, Treatments

## Abstract

**Background:**

The purpose of case report was to present a rare case of a solitary metastasis of myxoid liposarcoma and discuss the clinical and pathological information for patients treated for metastatic myxoid liposarcoma.

**Case presentation:**

We report our experience with a case of solitary metastasis of myxoid liposarcoma from the thigh to intraperitoneum. The patient was a 60-year-old man who was referred for abdominal discomfort and fatigue. Enhanced computed tomography showed a 25-cm intra-abdominal tumor. He had undergone a wide local excision for a right thigh myxoid liposarcoma 6 years earlier. At laparotomy, a huge multi-lobular cystic mass was identified at the small bowel mesentery. Wide local excision was performed, and the mass was diagnosed as metastatic myxoid liposarcoma. He was discharged without postoperative complications.

**Conclusions:**

We experienced a single intraperitoneal metastasis in a patient with myxoid liposarcoma after radical surgery of the primary site.

## Background

Liposarcoma is a mesenchymal malignant tumor arising from adipose tissue. Myxoid/round-cell liposarcoma (myxoid liposarcoma) is one of the major subtypes of liposarcoma characterized by lipomatous differentiation with a myxoid stroma and accounts for 30–40% of all liposarcomas [1, 2]. Myxoid liposarcoma occurs mainly in the lower extremities, followed by the retroperitoneum and trunk. Unfortunately, approximately 10% of patients with myxoid liposarcoma experience metastatic tumors, which occur in unusual locations, such as thorax and retroperitoneum [3]. The treatment method for myxoid liposarcoma is wide local excision of the tumor confirmed by negative resection margins. The decision to give adjuvant radiotherapy and chemotherapy is based on the risk of recurrence with metastatic disease [4]. We present a rare case of a 60-year-old man who suffered a solitary metastasis of myxoid liposarcoma from the thigh to intraperitoneum. We review the relevant literature and discuss feasible methods of treatment for this disease.

## Case presentation

**Fig. 1:**
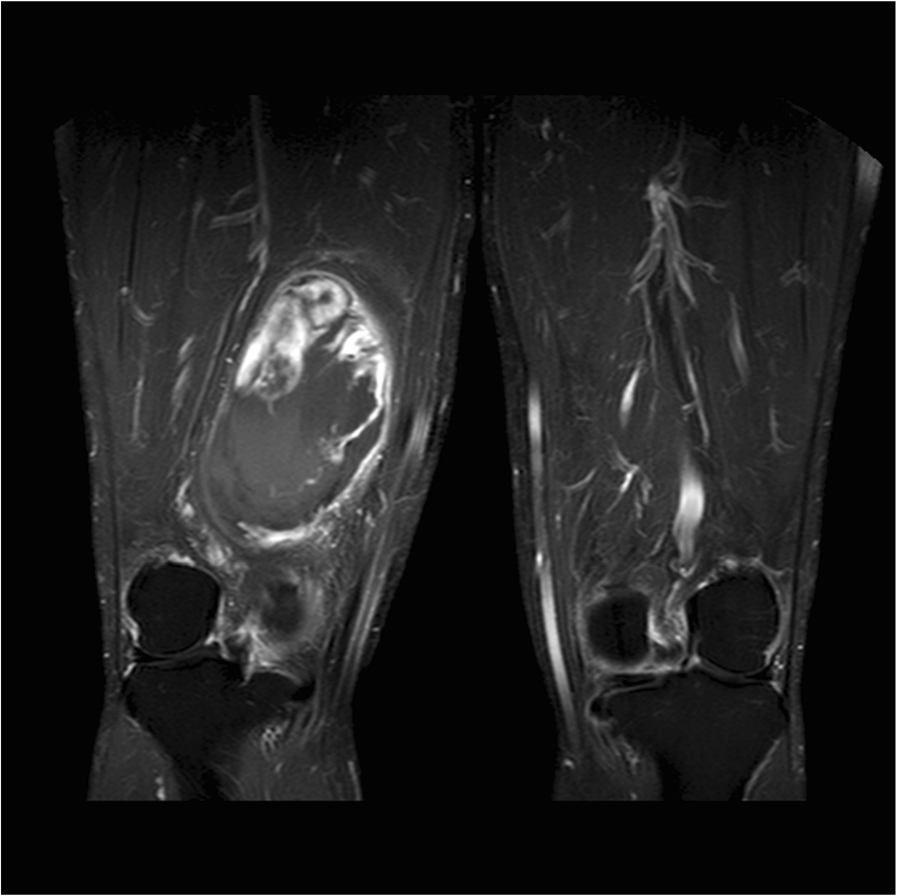
Magnetic resonance T2-weighted image on lower extremity

The patient was a 60-year-old male who was referred to our institution for an intra-abdominal lesion. Abdominal computed tomography performed at a local hospital revealed a huge intra-abdominal mass. The patient had symptoms of vague abdominal discomfort and fatigue, which persisted for > 4 months. Six years earlier, he had visited an orthopedic clinic at another institution with a chief complaint of a painless mass in his right thigh. He was suspected of having a liposarcoma by clinical magnetic resonance imaging (Fig. 1). The patient received wide local excision of the mass, and the maximum tumor diameter was 11 cm. The final pathological findings were myxoid liposarcoma with negative resection margins, stage IIIB according to the AJCC 8th edition [5]. The immunohistochemical findings were S-100(−) and CD34(−), and the positive rate of Ki67 was 1–2%. The patient was treated with adjuvant radiotherapy (200 cGy, 30 times), but lost up to follow-up after 2015. He had no significant medical or family history.

**Fig. 2:**
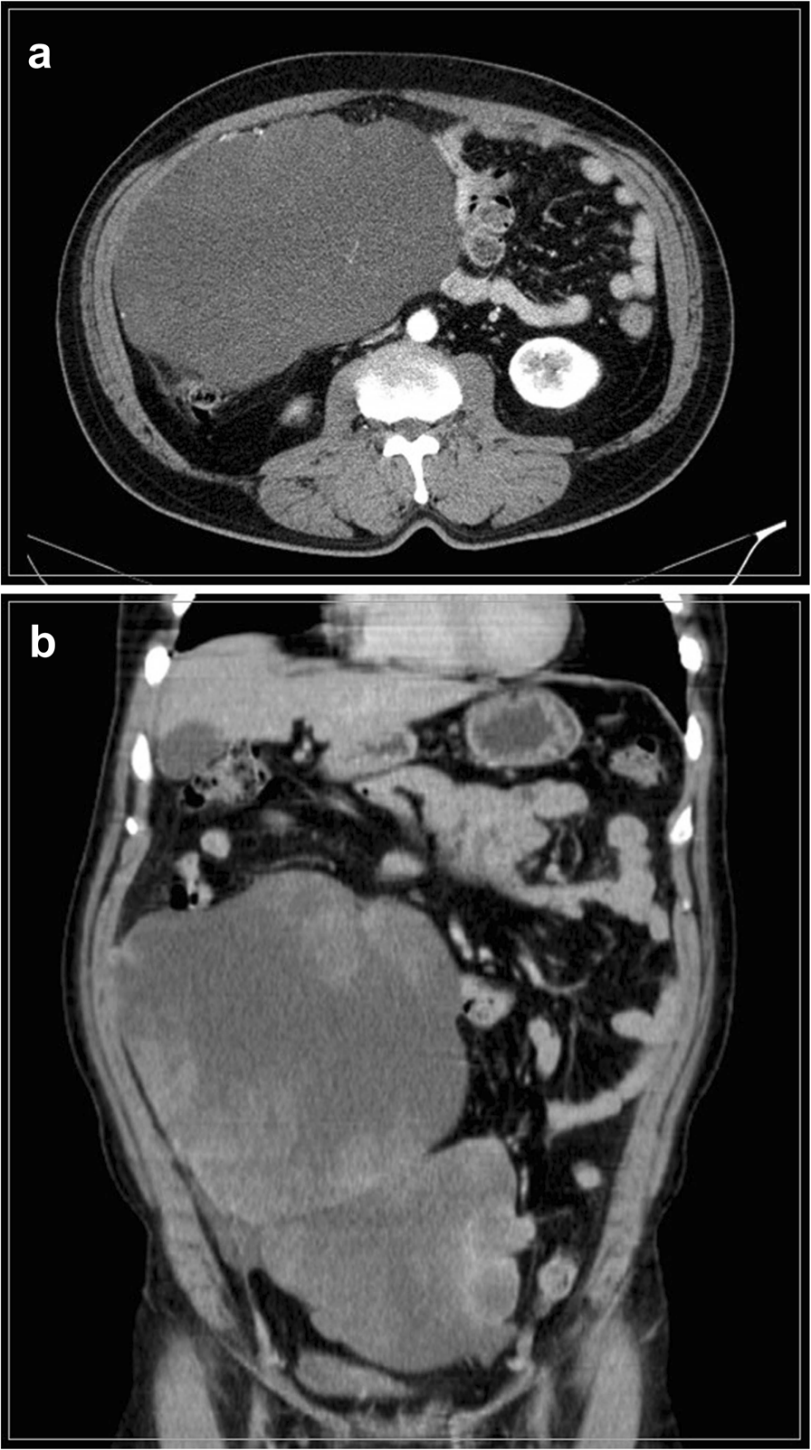
Computed tomography scan showing the presence of a huge mass lesion in the right mid to lower abdomen extending to the pelvic cavity. **a** Coronal view. **b** Sagittal view

**Fig. 3:**
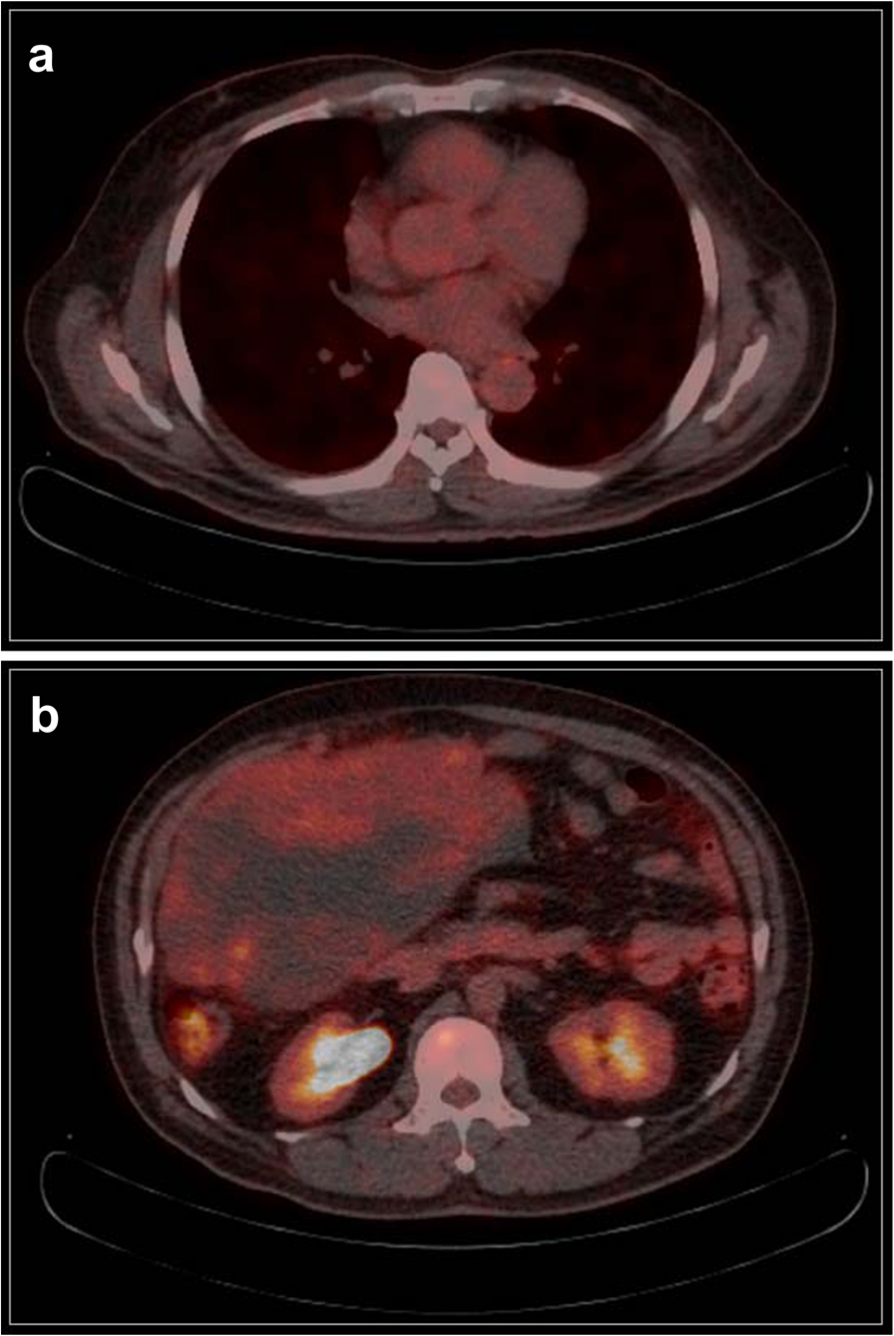
Positron emission tomography showing no distant metastasis. **a** Lung. **b** Abdomen

On physical examination, his blood pressure was 130/90 mmHg, heart rate was 90 beats/min, body temperature was 36.8 °C, respiratory rate was 20 breaths/min, and oxygen saturation at room air was 100%. The abdomen was moderately distended and rigid, without signs of peritonitis. A computed tomographic scan of his abdomen confirmed the presence of a heterogeneous enhancing mass lesion in the right mid-to-lower abdomen extending to the pelvic cavity (Fig. 2). Positron emission tomography showed no distant metastasis, including in both lung fields (Fig. 3). Core needle biopsy was not performed owing to the history of myxoid liposarcoma [6], and we decided to perform an exploratory laparotomy for both diagnosis and treatment. His breath sounds were normal, and there were no significant findings from review of other systems. The results from laboratory examination were normal, including those for blood chemistry, routine blood tests, and tumor markers.

**Fig. 4:**
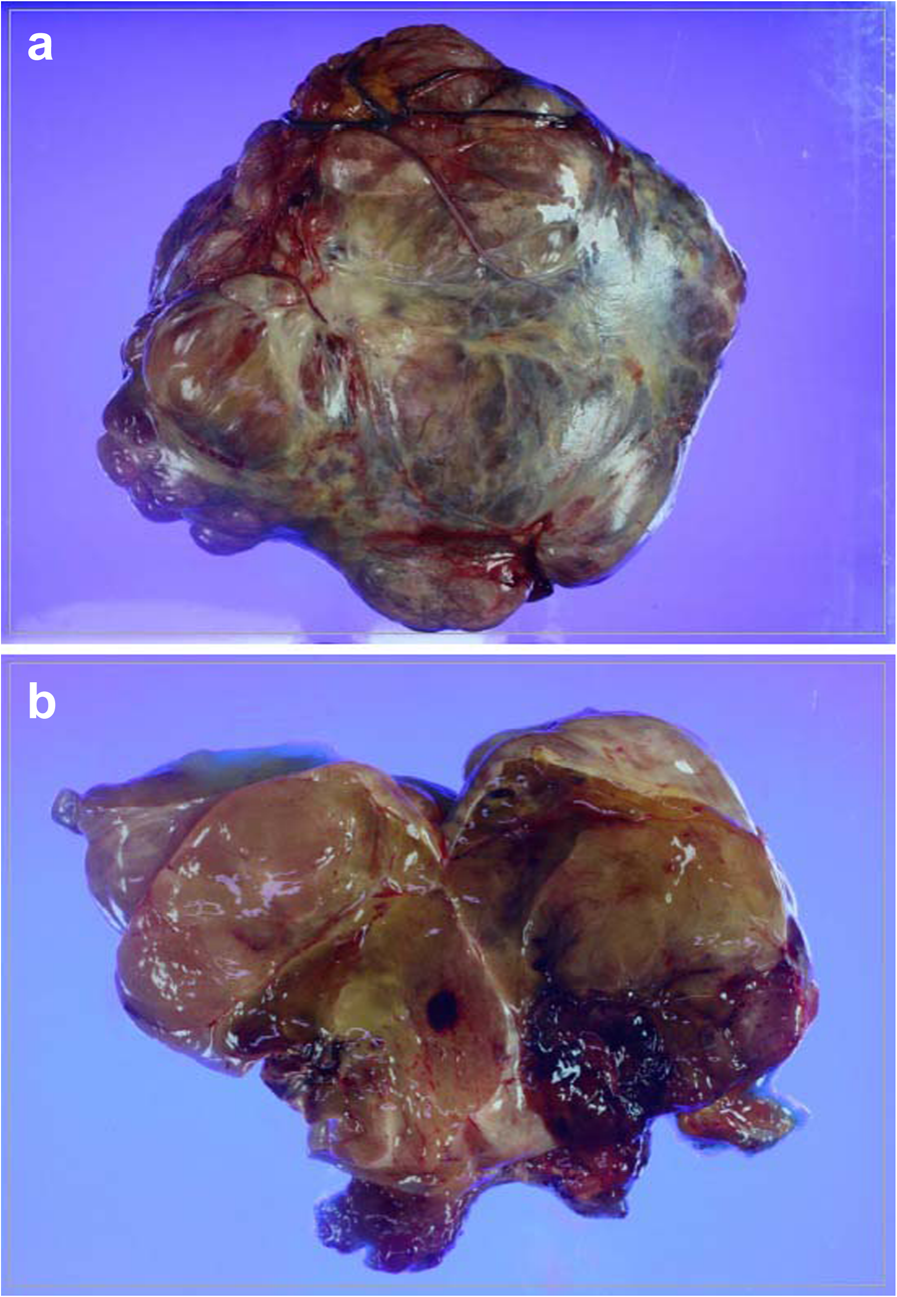
Gross appearance of resected specimen. **a** Tumor. **b** Cut section

The patient was taken to the operating room for an exploratory laparotomy. A mid-line abdominal incision was made, and there was no evidence of ascites and peritoneal or distant metastasis in the abdominal cavity. The tumor was a multi-lobulated gelatinous mass (Fig. 4) originating from the small bowel mesentery, 25 × 20 cm in size, so we performed wide local excision including small bowel resection and anastomosis. The patient was transferred to the general ward after operation and discharged 8 days after the surgery without immediate complications. At a follow-up appointment, the patient was in good condition without any symptoms and was transferred to a medical oncologist for further treatment. He is currently undergoing doxorubicin-based chemotherapy (75 mg/m^2^, every 3 weeks, eight cycles).

**Fig. 5:**
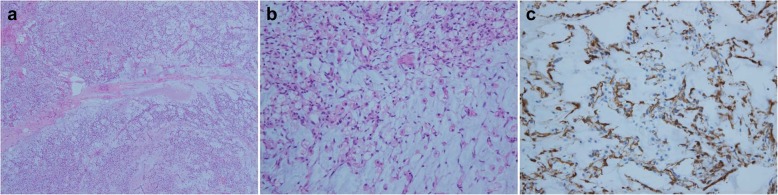
Histopathological examinations of metastatic myxoid liposarcoma. HE, hematoxylin and eosin-stained sample. **a** Low-degree malignant spindle cell tumor combined with mucoid degeneration, HE (× 40). **b** Uniform round-shaped non-lipogenic mesenchymal cell, HE (× 200). **c** Negative for lipoblast and lipogenic cell, CD34 (× 200)

Histopathological examination of the intra-abdominal tumor revealed paucicellular with monomorphic, stellate, or fusiform-shaped cells in the myxoid parenchyma that were diagnosed as metastatic myxoid liposarcoma (Fig. 5). Expert pathologist with 15 years of experience described the pathologic results for this study. The immunohistochemical findings were S-100(−), CD34(−), p53(−), desmin(−), SMA(−), EMA(−), CD31(−), HMB45(−), PanCK(−), and vimentin(+), and the positive rate of Ki67 was 5–7%. Finally, translocation t (12;16)(q13;11.2) FUS-DDIT3 was demonstrated by molecular analysis to confirm diagnosis of myxoid liposarcoma.

## Discussion and conclusions

Liposarcoma is classified into three different subtypes according to the molecular and clinicopathological differences. The World Health Organization updated contents of liposarcoma at sections of bone and soft tissue sarcoma in 2013 to include well-differentiated or de-differentiated liposarcoma, pleomorphic liposarcoma, and myxoid/round-cell liposarcoma [7]. Myxoid liposarcoma is the second most common type of liposarcoma and presents with slow growing patterns; however, it has aggressive clinical appearances similar to pleomorphic liposarcoma. Crago et al. reported that there is a 15% risk of local recurrence in 3 years and 10% of patients developed metastatic disease after wide local excision of the primary site [1].

While the lower extremity is the most common site, these tumors can occur in any extremity, intrathoracic sites, or abdominal sites [4]. Most patients have no typical symptoms at the tumor site, so this tumor is found to be fairly large at the time of diagnosis [8]. In this case, the patient actually visited an orthopedic clinic with a complaint of pain in his right ankle. He had no symptoms of right thigh lesion, and the physician noticed a large mass on simple X-ray imaging.

Imaging tests are essential for making detailed plans for surgery and finding distant metastases in myxoid liposarcoma. First, computed tomography (CT) or magnetic resonance imaging (MRI) should be performed to detect fatty tissue in the tumor. Myxoid liposarcoma has typical radiographic features that are attributed to the non-enhancing myxoid matrix of the tumor in contrast studies [9, 10]. Especially, MRI can show the presence of tumor necrosis, bone invasion, and encasement of vessels, which may indicate a high grade and poor prognosis. For staging work-up, CT of the abdomen and chest also should be performed to detect metastatic tumors. After a mass has been confirmed by imaging tests, percutaneous core needle biopsy or fine needle aspiration can be performed for accurate diagnosis [6].

Multidisciplinary team management has an important role in the treatment of myxoid liposarcoma. Surgery, radiation therapy, and systemic chemotherapy can be organically performed according to the staging [4, 11]. Several studies have demonstrated that multidisciplinary management reduced the rates of local recurrence [11, 12]. From the diagnosis to treatments, soft tissue sarcoma should be managed by a multidisciplinary team at a center with much experience. Surgery is the mainstay treatment of myxoid liposarcoma, and the standard procedure is wide local excision with negative resection margins of ≥ 10 mm of adjacent tissue [13]. Function-preserving surgery is the best option for limb-sparing and quality of life, but amputation should be considered occasionally in case of deep invasion into adjacent organs.

Radiation therapy helps to lower the risk of local recurrence and metastasis [4, 11]. Myxoid liposarcoma is more radiosensitive than other types of soft tissue sarcomas [11]. Research has continued on whether neoadjuvant is more advantageous than adjuvant radiation with regard to clinical outcomes. Radiation therapy has been proven to be as effective as adjuvant therapy in terms of obtaining local control, especially in patients with high grade, deep lesions of > 5 cm [4]. Neoadjuvant or adjuvant chemotherapy may be one of the treatment options for patients with myxoid sarcoma, but the evidence remains controversial. Anthracycline-based or doxorubicin-based chemotherapy has been considered to be the standard treatment for chemotherapeutic agents. The decision to give adjuvant chemotherapy should be based on the risk of recurrence with metastatic disease, and high-risk soft tissue sarcomas warrant consideration of adjuvant chemotherapy [13–15]. Tumor size, depth, site, age, and histology (round cell percentage in myxoid liposarcoma) are included to calculate the risk of soft tissue sarcoma [4].

Myxoid liposarcoma is likely to show both local recurrence and distant metastasis. Unlike other types of liposarcoma, myxoid liposarcoma presents an uncommon tendency of extrapulmonary metastasis. Several studies have reported that the common metastatic sites were the retroperitoneum, extremities, thorax, and subcutaneous soft tissue [8, 16]. In abdominal areas, intraperitoneal metastasis is rare compared with retroperitoneum metastasis, which is represented by the adrenal gland and pancreas. Furthermore, there have been some case reports of other unusual sites of metastasis [17–19], but metastasis to the small bowel mesentery is very rare. To the best of our knowledge, our case is the first report about solitary metastasis of myxoid liposarcoma from the thigh to small bowel mesentery in Korea. Although intraperitoneal metastasis is rare, its clinical meaning is still not clear. Further studies are needed to reveal the hypothesis of the different routes of metastasis other than retroperitoneum.

Several studies have reported that an R0 resection can increase the long-term survival rate and a metastasectomy could significantly improve prognosis in patients with metastasis [19, 20]. In our case, the primary tumor had already been resected, and disease-free status was obtained by wide local excision, which could improve survival in myxoid liposarcoma. However, established treatment methods have not been standardized for the treatment of metastatic myxoid liposarcoma. For curing metastatic myxoid liposarcoma, other treatment methods have been performed occasionally, including doxorubicin-based chemotherapy, trabectedin, eribulin, tyrosine kinase inhibitors, and molecular-targeted therapy [4]. We decided to perform wide local excision in this case, but it is still unclear which combination of treatments will work best. A well-designed study is needed to determine which choice will yield the best prognosis.

In conclusion, we experienced a single intraperitoneal metastasis in a patient with myxoid liposarcoma after wide local excision of the primary site. Radical metastasectomy may be optimal for the treatment of a single metastasis in the intraperitoneal organs. Even though intraperitoneal metastasis of myxoid liposarcoma is very rare, we should consider the possibility of intraperitoneal metastasis in patients with myxoid liposarcoma in whom a growing mass in the abdomen is observed.

## Data Availability

All data generated or analyzed during this study are included in this published article.
